# A dedicated phantom for exploring the interplay of fat and paramagnetic substances in quantitative susceptibility mapping

**DOI:** 10.1007/s10334-025-01261-3

**Published:** 2025-06-02

**Authors:** Simon Graf, Josefine Trapp, Maik Rothe, Alexander Gussew, Walter A. Wohlgemuth, Andreas Deistung

**Affiliations:** 1 University Clinic and Outpatient Clinic for Radiology, University Hospital Halle (Saale), Ernst-Grube-Str. 40, 06120 Halle (Saale), Germany; 2 Halle MR Imaging Core Facility, Medical Faculty of Martin-Luther-University Halle-Wittenberg, Ernst-Grube-Str. 40, 06120 Halle (Saale), Germany

**Keywords:** Quantitative susceptibility mapping, Relaxometry, Phantom, Water–fat separation, Iron

## Abstract

**Objective:**

Accurate quantitative tissue characterization in organs with considerable fat content, like the liver, requires thorough understanding of fat’s influence on the MR signal. To continue the investigations into the use of quantitative susceptibility mapping (QSM) in abdominal regions, we present a dedicated phantom that replicates liver-like conditions in terms of effective transverse relaxation rates (*R*_2_*) and proton density fat fractions.

**Materials and methods:**

The spherical agar phantom consists of nine smaller spheres (diameter: 3 cm) doped with a paramagnetic substance (iron nanoparticles or manganese chloride) and fat (peanut oil), embedded in a large agar sphere (diameter: 14 cm), ensuring no barriers exist between the enclosed spheres and their surrounding medium. Concentrations were selected to represent both healthy and pathologic conditions. 3T MRI measurements for relaxometry, fat–water imaging, and QSM were conducted with the head coil and for ^1^H-spectroscopy with the knee coil at three time points, including a scan–rescan assessment and a follow-up measurement 14 months later.

**Results:**

The phantoms’ relaxation and magnetic properties are in similar range as reported for liver tissue. Substantial alterations in local field and susceptilibty maps were observed in regions with elevated fat and iron content, where fat correction of the local field via chemical shift-encoded reconstruction effectively reduced streaking artifacts in susceptibility maps and substantially increased susceptibility values. Linear regression analysis revealed a consistent linear relationship between *R*_2_* and magnetic susceptibility, as well as iron concentration and magnetic susceptibility. The relaxation, fat, and susceptibility measurements remained stable across scan–rescan assessment and long-term follow-up.

**Discussion:**

We developed a versatile phantom to study fat–iron interactions in abdominal imaging, facilitating the optimization and comparison of susceptibility processing methods in future research.

**Supplementary Information:**

The online version contains supplementary material available at 10.1007/s10334-025-01261-3.

## Introduction

The human body is composed of numerous tissue types, each with distinct characteristics and functions, and relies on multiple essential elements. Iron, for instance, is crucial for oxygen transport and DNA synthesis [1] and is simultaneously under strict regulation, since excess iron can lead to various iron-related disorders such as iron overload [2] or neurodegenerative diseases [3, 4]. The total body iron level is typically assessed with the plasma ferritin marker; however, it is affected by conditions such as acute infections or chronic inflammation, altering the relationship between plasma ferritin and total iron levels [5, 6]. In the human liver, iron ions (Fe^3+^) are stored primarily in the cores of ferritin shells and hemosiderin [7, 8] and its iron content is directly linked to the total amount of iron in the body [9]. Therefore, measuring liver iron content (LIC) is commonly seen as the most accurate way to evaluate the total body iron content. LIC has historically been assessed using liver biopsy and destructive biochemical analysis [10]. However, the clinical utility of liver biopsy for LIC quantification is limited due to its invasive nature and substantial sampling variability [11–13], making it unsuitable for repeated measurements required for treatment monitoring. As a result, non-invasive methods, such as magnetic resonance imaging, have largely replaced biopsy for LIC assessment. Determination of the transverse relaxation rate (*R*_2_) or effective transverse relaxation rate (*R*_2_^*^) in conjunction with dedicated calibration curves are the most widely used MRI approaches for quantifying LIC [14, 15]. However, these MRI-based estimations of LIC are prone to inaccuracies arising from breathing motion, and especially the presence of abdominal adipose tissue [13, 16], as well as discrepancies in the LIC versus *R*_2_/*R*_2_^*^ calibration curves with respect to the used acquisition and processing parameters [17]. Direct measurement of tissue magnetic susceptibility can offer advantages over MRI relaxometry for LIC measurement. While *R*_2_^*^ is influenced by the microscopic distribution of iron, the magnetic susceptibility remains unaffected [18, 19]. Moreover, magnetic susceptibility measurements have the capability to circumvent the necessity for calibration curves, providing a direct correlation with LIC [20, 21].

A way to derive the magnetic susceptibility distribution in vivo using MRI is possible by applying quantitative susceptibility mapping (QSM) [22, 23]. The method relies on sophisticated image processing of raw gradient-recalled echo phase images and has been conducted successfully across various regions of the brain to investigate iron deposits [24–26], myelination [27], microbleeds, and venous vasculature [28, 29] and to differentiate between iron and calcium deposits [30, 31]. While QSM has demonstrated its utility and reliability across a wide range of neurological and neurodegenerative applications, its use beyond the central nervous system remains limited, with only initial applications in the heart, liver, prostate, and cartilage [32]. In this context, susceptibility-based characterization of abdominal organs, particularly the liver, is of special interest as it offers an alternative method for measuring LIC. A few studies have already been applied to investigate hepatic iron content [33–35] and hepatic fibrosis [36, 37] using QSM.

In contrast to brain tissue, abdominal tissue is characterized by substantial contributions of fat. The coexistence of fat and water leads to signal intensity oscillations due to the constructive and destructive interference of these two components during multi-echo gradient-echo acquisitions. Furthermore, adipose tissue induces chemical shifts, which introduce non-susceptibility-related contributions to the gradient-echo phase data. This phase data is used in QSM to derive magnetic field maps, which are subsequently employed in the field-to-susceptibility inversion process. The presence of non-susceptibility-related phase contributions can increase the likelihood of artifacts, such as streaking, in susceptibility maps. To mitigate these effects, abdominal QSM studies frequently employ water–fat separation techniques as a preprocessing step to correct for fat-related contributions [33–35].

In quantitative MRI, a dedicated phantom facilitates the precise evaluation of developed methods [38], which in our case considers the influence of fat and subsequent fat-correction techniques on local field and susceptibility maps to enhance the reliability of LIC quantification. Zhao et al. [39] replicated the presence of fat, iron, and fibrosis and performed comprehensive relaxometry and proton density fat fraction (PDFF) evaluations. Specifically targeting QSM, Li et al. [19] built a phantom that embedded balloons, filled with gadolinium (as paramagnetic substance), fat, collagen, and water, in a water-filled container to perform *R*_2_* and susceptibility mapping. Kim et al. [40] proposed a spherical gelatin phantom, in which gelatinous inclusions of iron oxide-based contrast agents as paramagnetic source were integrated, to evaluate QSM processing. The main limitation of the phantoms proposed in [19] 39 for QSM processing is their use of plastic vials as vessel for the different solutions embedded in a water-filled container. While suitable for relaxometry and the advantage of a relatively straightforward assembly, the plastic interfaces of the vials can induce Gibbs ringing and magnitude signal loss, which may impede consistent phase unwrapping (e.g., when using region-growing approaches) or amplify non-local artifacts if not properly addressed. The phantom presented in [40] demonstrates the feasibility of designing a spherical phantom containing inclusions without an additional interface to the surrounding medium; however, the *R*_2_* values are below 23 s^−1^ at 3T, mimicking LIC only within the healthy range [41]. In addition, the authors did not include a fat source.

Inspired by the works of Kim et al. [40] and Zhao et al. [39], we developed a novel spherical phantom simulating both healthy and pathological liver tissue, specifically targeting abdominal QSM processing, which mimics both healthy and pathological liver tissue. We removed plastic interfaces in the phantom and embedded nine different solutions of agar base doped with fat and iron nanoparticles within an enclosed agar sphere. The phantom was comprehensively characterized using relaxometry (*R*_1_, *R*_2_, *R*_2_^*^), water–fat imaging, ^1^H-MR spectroscopy, and QSM (single- and multi-orientation), including a 14-month follow-up measurement and a scan–rescan assessment. This work focuses on the design, construction, and reproducibility characterization (short-term reproducibility and long-term stability) of the phantom, to assess its feasibility for relaxometry and QSM processing. Additionally, we explored the use of manganese chloride as a paramagnetic substance as a potential cost-effective alternative to iron nanoparticles.

## Theory

The complex-valued MR signal obtained with gradient echo measurements in body parts containing water and lipid components is well established. Here, we briefly introduce the relationship between gradient-echo phase images, magnetic susceptibility, and the chemical shift in adipose tissue.

The complex-valued MR signal within the voxel obtained with gradient echo measurements in body parts containing water and lipid components is given by [42]:

1 $$S\left({\rho }_{W}, {\rho }_{F},{f}_{B},{R}_{2}^{*}\right)=\left({\rho }_{W}+ {\rho }_{F}\sum_{p=1}^{P}{\alpha }_{p}{e}^{i2\pi {f}_{F,p}t}\right) {e}^{i({\phi }_{0}+2\pi {f}_{B}t)}{e}^{-{R}_{2}^{*}t},$$

where ${\rho }_{W}$ and ${\rho }_{F}$ are the amplitudes of the water and lipid signal, respectively, ${\phi }_{0}$ is the initial phase introduced by the radiofrequency response of the sample, ${f}_{B}$ is the frequency shift due to field inhomogeneities, e.g., due to susceptibility effects or imperfect shimming, ${R}_{2}^{*}$ is the effective transverse relaxation rate of the voxel, $p$ is the number of lipid peaks, ${f}_{F,p}$ is the lipid peak frequencies relative to the water peak, ${\alpha }_{p}$ is the relative amplitude of the lipid peaks ($\sum_{p-1}^{P}{\alpha }_{p}=1$), and $t$ is the echo time. The lipid peak frequencies ${f}_{F,p}$ and the corresponding amplitude ${\alpha }_{p}$ may be determined using ^1^H-MR spectroscopy to reduce the number of unknowns when solving for ${\rho }_{W}$, ${\rho }_{F}$, ${f}_{B}$, and ${R}_{2}^{*}.$ In case only the magnitude is available, the phase-dependent term in Eq. 1 can be discarded while accounting for the lipid-induced contribution based on the previously determined amplitudes and frequencies of the lipid peaks. When interested in magnetic susceptibility, magnitude and phase data have to be considered in Eq. 1.

The gradient echo phase $\phi \left(\overrightarrow{r}, t\right)$ is given by [22, 43]:

2 $$\phi \left(\overrightarrow{r}, t\right)= {\phi }_{0}\left(\overrightarrow{r}\right)+ 2\pi\Delta f\left(\overrightarrow{r}\right)t  \approx\phi_0\left(\vec{r}\right)+\gamma\left(\left(\frac{2\pi}{\gamma}f_B\left(\vec{r}\right)\right)\left(1-\sigma\left(\vec{r}\right))-B_0\sigma(\vec{r})\right)\right)t$$

with ${\phi }_{0}\left(\overrightarrow{r}\right)$ being the initial phase [44] (see Eq. 1), $\Delta f\left(\overrightarrow{r}\right)$ the measured frequency variation, $\gamma$ the gyromagnetic ratio, ${f}_{B}\left(\overrightarrow{r}\right)= \frac{\gamma }{2\pi }({B}_{inh}\left(\overrightarrow{r}\right)+{B}_{\chi }\left(\overrightarrow{r}\right))$ (see Eq. 1), $\sigma \left(\overrightarrow{r}\right)$ the chemical shift [45], and $\overrightarrow{r}$ the spatial location vector. With respect to Eq. 1, $\sigma$ denotes the spectral peak area-weighted average chemical shift that can be defined as $\sigma =\overline{{f }_{F}}$ in the voxel. Chemical shift encoded reconstruction incorporating fat correction [46–48] effectively eliminates the spatially varying field contributions arising from the chemical shift, $\sigma \left(\overrightarrow{r}\right)$. The magnetic susceptibility $\chi (\overrightarrow{r})$ can be extracted from the phase data via deconvolution with the unit dipole response ${d}_{z}(\overrightarrow{r})$, in case of appropriate shimming, and if magnetic field contributions arising from magnetic sources outside a defined volume of interest (VOI, e.g., the area of the brain if interest in brain imaging) ${B}_{\chi , \text{ext}}\left(\overrightarrow{r}\right)$ are removed properly, e.g., with sophisticated harmonic artifact removal for phase data (SHARP) [49], only the sources within the VOI ${B}_{\chi , \text{int}}(\overrightarrow{r})$ contribute:

3 $$\tilde{B}\left( {\vec{r}} \right) \approx B_{0} ~\left( {{{\chi }}\left( {\vec{r}} \right) \otimes d_{z} \left( {\vec{r}} \right)} \right).$$

Since the phase is influenced by the chemical shift due to lipid sources (Eqs. 1 and 2), accurate fat correction is essential to determine the susceptibility-induced magnetic field map [32]. A detailed derivation of Eqs. 2 and 3 is provided in the supplementary.

To this day, the COSMOS approach (calculation of susceptibility through multiple orientation sampling) is the gold standard method for QSM measurements [50]. While its feasibility in a clinical setting is already limited for brain MRI, it is barely possible for abdominal measurements. For phantom measurements, however, it provides a viable opportunity to measure and compute ground truth susceptibility data.

## Material and methods

To investigate the impact of fat on the susceptibility map, a comprehensive multi-purpose phantom was constructed. Typical proton density fat fractions (PDFF) values range from under 5% for healthy tissue to over 30% in patients with steatosis grade 3 [51–53]. For hepatic iron overload, healthy *R*_2_* values are below 126 s^−1^, increasing to approximately 240 s^−1^, 1160 s^−1^, or even higher for mild, moderate, and severe overload, respectively [41, 54]. The phantom design was based on the following prerequisites: (i) inclusion of fat and iron sources simulating liver tissue, (ii) *R*_2_^*^ rates and PDFF within healthy to mildly pathologic ranges (PDFF up to 20%, *R*_2_* up to 250 s^−1^), (iii) spherical design allowing high reproducibility, and (iv) absence of a structural barrier between the different compositions.

## Spherical agar phantom

**Fig. 1:**
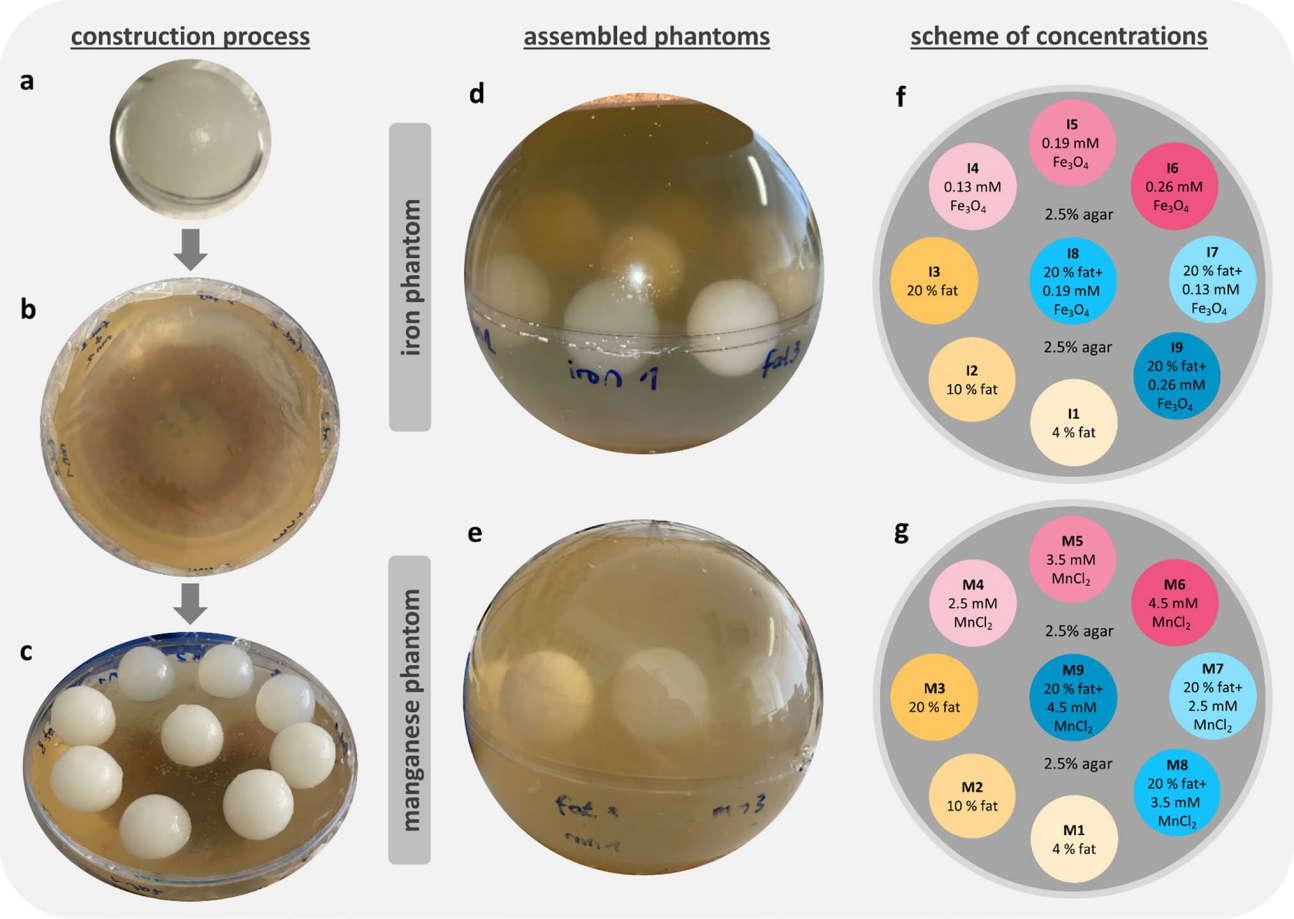
Phantom construction process and schematic representation of the phantoms. The small spheres are prepared in small polystyrene shells (**a**). The lower hemisphere is coated with cling foil during the cooling process (**b**). After removing the small spheres from their shells, they are placed on the lower phantom hemisphere (**c**), the outer shell is closed and filled with agar to the rim for the iron (**d**) and manganese phantoms (**e**). Both spherical phantoms differ only in the used paramagnetic substance, iron nanoparticles, (**f**) and manganese chloride (**g**). The spheres are numbered from 1 to 9, with each number corresponding to an increasing concentration of fat, paramagnetic substance, or both

The generated spherical phantoms are illustrated in Fig. 1 and consist of nine small agar spheres doped with varying amounts of paramagnetic substances and fat. The base solution used for phantom construction was a 2.5 wt. % water–agar solution (agar–agar powder, Carl Roth GmbH + Co. KG) for the large sphere (diameter of 14 cm), and 2 wt. % water–agar solution for the small spheres (diameter of 3 cm). The phantom size of 14 cm approximates the average liver diameter (male: 14 cm, female: 14.5 cm) [55]. Sodium azide, 0.1 wt. % (Sigma-Aldrich, Merck KGaA), was used to preserve the phantom and prevent contamination (e.g., bacteria or mold). A coating agent (Imprägnol, Brauns-Heitmann GmbH & Co. KG) was used to prevent diffusion of water between the different phantom areas, maintaining the adjusted concentrations. To increase the melting point of the small spheres, prevent melting and maintain the different volume integrities, 1% formaldehyde (CH_2_O) (Carl Roth GmbH + Co. KG) was added to the small spheres. The contrast agent Gadovist (1 mmol mL^−1^, Bayer Vital GmbH) was used to modulate the longitudinal relaxation rate in the large and small spheres. Iron nanoparticles (5mg mL^−1^ iron oxide (II, III), magnetic nanoparticle solution, Sigma-Aldrich, Merck KGaA) or MnCl_2_ (manganese(II) chloride monohydrate, Carl Roth GmbH + Co. KG) were used as paramagnetic substances to elevate *R*_2_^*^. The spherical phantom with iron nanoparticles is herein referred to as the iron phantom, while the one with MnCl_2_ is referred to as the manganese phantom. As fat source, peanut oil (Kunella Feinkost GmbH, Cottbus, Germany) was chosen, since the proton NMR spectrum is similar to that of human adipose tissue [56]. The adjusted concentrations for the different phantom parts are given in Table 1.

**Table 1:** Composition of the spherical iron and manganese phantom

Volumes			Both phantoms			Iron phantom			Manganese phantom		
			Gadovist [mM]	NaN_3_ [wt%]	CH_2_O [vol%]	Peanut oil [vol %]	Agar [wt %]	Fe_3_O_4_ [mM]	Peanut oil [vol %]	Agar [wt %]	MnCl_2_ [mM]
Small sphere	Lipid only	S1	0.25	0.1	1	4	2		4	2	
		S2				10			10		
		S3				20			20		
	Agent only	S4				0		0.13	0		2.5
		S5						0.19			3.5
		S6						0.26			4.5
		S7				20		0.13	20		2.5
		S8						0.19			3.5
		S9						0.26			4.5
Big sphere			0.25	0.1	0	0	2.5	0	0	2.5	0

## Phantom assembly

For the construction of the phantom, nine vials (S1–S9) were used to prepare the individual solutions. Different amounts of paramagnetic substances (iron nanoparticles or MnCl_2_) were added by pipetting to the vials (S4–S9). A manganese solution was prepared with water, whereas the iron nanoparticles were already delivered in a solution. Formaldehyde was added to all vials. The water–agar base solution was prepared with distilled water, Gadovist, and sodium azide. The components were heated to approximately 90 °C with constant stirring. The agar powder was added to the solution and stirred until fully dissolved. Peanut oil was added to a beaker and stirred until emulsification, indicated by the solution turning white. Four different base solutions (2wt. % agar with 0, 4, 10, and 20% fat) were necessary for the small spheres of the phantom. The base solution was added to the respective vials and stirred for 15 s on a vortex to ensure proper mixing. After that, the solutions were poured into the small spheres. Once cooled, the small spheres were removed from the polystyrene shells, sprayed with the coating agent, and left to dry for approximately 24 h (Fig. 1a). On the next day, the large sphere was constructed in two steps: first, the lower hemisphere (Fig. 1b, c) and then the final phantom assembly (Fig. 1d, e). A 2.5 wt. % base solution was prepared and half of the shell of the lower hemisphere was filled. A cling foil was used to cover the drying agar solution to prevent film formation (Fig. 1b). Preliminary studies showed the formation of artifacts at the interhemispheric interface. Thus, the lower shell was only filled half, to move the interhemispheric interface under the small spheres. While the base solution was left to cool for approximately 30 min in the lower hemisphere shell, the 2.5 wt. % base solution for the final phantom assembly was prepared. To minimize the formation of an interface, the base solution of the lower hemisphere should not be fully gelled. The cling film was removed and the small spheres were placed onto the agar solution (Fig. 1c). The spherical shell was closed with the upper lid and the sphere filled to the rim with base solution through a hole in the shell of the upper hemisphere (Fig. 1d, e). The phantom was left to cool at least for 24 h before conducting the MR measurements. The phantoms were stored in a container to minimize air contact and the hole in the shell was closed. The construction process is identical for the two versions of the built phantom and only differs in the use of the paramagnetic substance.

## Data acquisition

Measurements were conducted on a 3T Siemens Magnetom Vida Scanner (Siemens Healthcare GmbH, Erlangen, Germany) with a 64-channel phased-array head coil. A 2D turbo spin echo inversion recovery (IR-TSE) sequence was used to collect data for *R*_1_ quantification and 2D multi-echo spin echo (ME-SE) imaging was employed to determine *R*_2_. A 3D multi-echo gradient-echo sequence (GRE) based on the volumetric interpolated breath-hold examination (VIBE) with six echoes was applied for water–fat separation, *R*_2_^*^ mapping, and QSM. Further data for QSM relying on COSMOS were acquired using the GRE-VIBE sequence in four additional different phantom positions with respect to the main magnetic field (${\overrightarrow{B}}_{0}$, tilts by approximately ± 60° in the $\overrightarrow{y}$-$\overrightarrow{z}$ plane and $\overrightarrow{x}$-$\overrightarrow{z}$, if $\overrightarrow{z}$ is the direction of the magnetic field). MR spectroscopic measurements were conducted with the stimulated echo acquisition mode sequence (STEAM) using a transmit/receive knee coil with 18 receive channels to determine the exact lipid spectrum in the phantom to parameterize ${f}_{F,p}$ and ${\alpha }_{p}$ in Eq. 1. The detailed sequence parameters are listed in Table 2. To assess repeatability (scan–rescan) and long-term stability, the phantom was additionally measured twice with an identical scanning protocol (except MR spectroscopy) 14 months after the initial phantom construction. For the scan–rescan assessment, the phantom was completely removed from the scanner and positioned a second time.

**Table 2:** Sequence parameters for the phantom measurements

Parameters	*R*_1_	*R*_2_	*R*_2_^*^ and PDFF	Spectroscopy
Sequence	IR-TSE	ME-SE	GRE-VIBE	STEAM
TR [ms]	10,000	3000	11.5	3000
TI [ms]	50, 150, 300, 600, 900, 1200, 1500	–	–	–
No. of echoes	1	14	6	7
TE [ms]	57	9.4	1.17	20, 30, 40, 50, 60, 100, 200
ΔTE [ms]		9.4	1.71	–
TM [ms]	–	–	–	10
Flip angle [°]	128	180	9	90
Voxel size [mm^3^]	1.0 × 1.0 × 2.0	0.8 × 0.8 × 4.0	0.8 × 0.8 × 0.8	14 × 14 × 14
Field of view [mm^3^]	192 × 192 × 88	250 × 250 × 60	190 × 194 × 128	–
Bandwidth [Hz px^−1^]	137	781	740	acq. BW.: 10 000 Hz
Acquisition time [min:s]	4:52 per TI	12:05	2:20	1:12 per spectrum
No. of spectra	–	–	–	6 per VOI

## Data processing

**Fig. 2:**
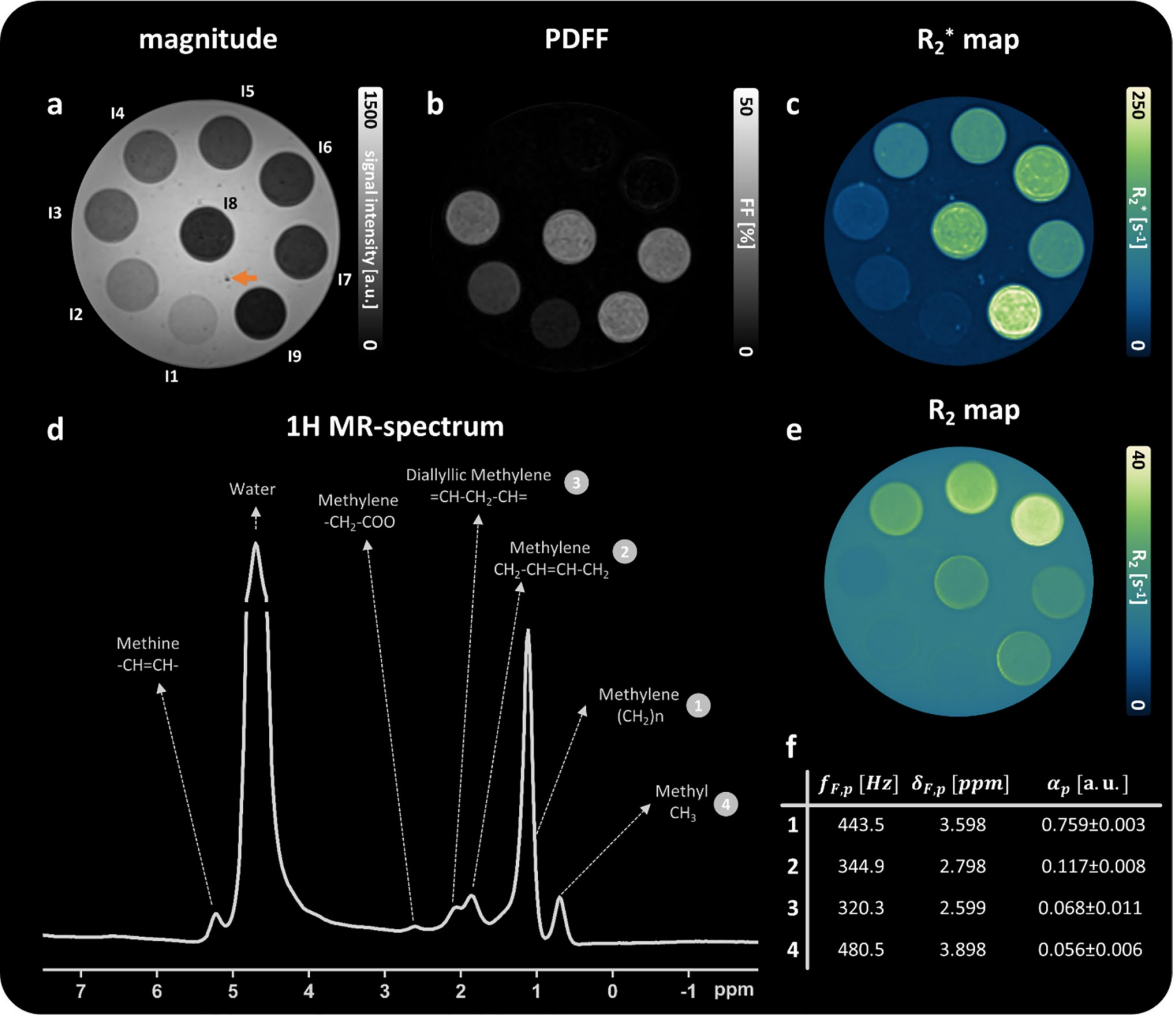
Comprehensive characterization of the iron phantom. One representative slice of the GRE-VIBE magnitude image at TE = 8.01 ms (**a**), the fat fraction map (**b**), the ${R}_{2}^{*}$ map (**c**), and *R*_2_ map (**e**) are presented. The proton MR spectrum (**d**, 6 averages) for inclusion I3 and the resulting fat characterizing parameters (lipid peak frequencies in Hz [${\text{f}}_{\text{F},\text{p}}$] and ppm [${\updelta }_{\text{F},\text{p}}$], relative amplitudes [${{\alpha }}_{\text{p}}$]) are shown in (**d**) and (**f**), respectively. The numbers adjacent to the individual lipid peaks in **d** refer to the numbers in **f**. The orange arrow in **a** indicates an air inclusion. The small spherical inclusions are exemplarily labeled in (**a**)

*R*_1_ was determined from the IR-TSE data sets with varying inversion times using ordinary least squares fitting [57] in a VOI-based manner. The monoexponential signal decay of the multi-echo spin echo data was fitted voxel-wise in an ordinary least squares manner to compute *R*_2_. Based on ^1^H-MR spectroscopy measurements, the intensities and resonance frequency of particular lipid compartments defining the lipid spectrum in Eq. 1 were obtained (Fig. 2f). All spectra were preprocessed and quantified using the jMRUI package [58, 59] (http://www.jmrui.eu). The relative amplitudes of the lipid spectrum ${\alpha }_{p}$ were calculated by dividing the area under the respective peak by the sum of the area under all lipid peaks and additionally corrected for fat compartment-specific *T*_2_ attenuations based on a monoexponential fit of signal decays in the STEAM TE series. The lipid peak frequencies ${f}_{F,p}$ were determined by multiplying the chemical shift difference of the lipid peaks and the water peak to the resonance frequency of the MR scanner (123.195 MHz). The parameters were computed for spectra acquired in the iron-free spheres with the two highest fat concentrations (inclusion I2 and I3 in Fig. 1f) and subsequently averaged. The complex-valued GRE-VIBE signal decay, acquired at intervals of 1.71 ms, beginning at 1.17 ms, was processed using water–fat separation to derive fat-corrected field maps for QSM [46], while *R*_2_* and PDFF were determined by fitting Eq. 1 to the magnitude signal decay only using a nonlinear least squares model, incorporating the phantom-specific lipid spectrum while disregarding ${\phi }_{0}$ and ${f}_{B}$ (see also section Theory, [42]).

To correct for fat-induced errors in the phase-derived magnetic field map, Eq. 1 was efficiently solved using the iterative graph-cut algorithm proposed by Hernando et al. [46], and applied to the complex-valued GRE-VIBE data. This technique jointly estimates the field map and water–fat images by discretizing the field map and applying an iterative binary graph-cut approach to optimize water–fat separation while avoiding local minima. Here, spatial smoothness constraints enhance robustness against field inhomogeneities while preserving local accuracy. The resulting fat-corrected field maps were then used for further susceptibility processing. For comparison, fat-uncorrected, wrapped multi-echo phase data were unwrapped [60], divided by ${TE}_{i}* \gamma$, and combined across echo times to generate fat-uncorrected field maps. Both field maps were further processed with sophisticated harmonic artifact removal for phase data [49] (SHARP) using ten different spherical kernels (1–10 voxels, regularized at a high-pass cutoff frequency of 0.02 mm^−1^) to compute the local magnetic field perturbation. The field-to-susceptibility inversion of the local field maps was conducted using homogeneity-enabled incremental dipole inversion (HEIDI) [61] for single-orientation inversion. COSMOS measurements were conducted to derive the magnetic susceptibility from multiple phantom orientations. The local field maps (corrected and uncorrected for fat contributions) were also computed for the four other GRE-VIBE acquisitions with varying phantom positions to ${\overrightarrow{B}}_{0}$ and then linearly transformed (6 degrees of freedom) using advanced normalization tools (ANTs [62], https://stnava.github.io/ANTs/) to the data set of the first measurement. Simultaneous inversion of the aligned local fields (5 data sets) to magnetic susceptibility was performed to derive the COSMOS maps. We referenced all susceptibility maps to the average susceptibility of 2.5% agar base solution (see Fig. 1f, g) and stated susceptibility values in parts-per-billion (ppb).

Volumes of interests (VOIs) were manually drawn in the nine spherical inclusions and their embedding medium for both the iron and manganese phantom using ITK-SNAP [63] (http://www.itksnap.org/pmwiki/pmwiki.php). Means and standard deviations were computed for the different VOIs. A one-way repeated measures ANOVA was performed separately for *R*_1_, *R*_2_, *R*_2_*, PDFF, and susceptibility values to assess statistical significance in repeatability and long-term stability. The analysis included the values of the nine different spherical inclusions measured across three time points.

## Results

### Phantom characterization

Parametric maps of the iron phantom and their VOI-based analysis are shown in Fig. 2 and Table 3, respectively. The high-resolution GRE magnitude image (Fig. 2a) shows a homogenous background in the large agar sphere with just a few dark speckles (orange arrow) air inclusions. The measured PDFFs agree well with the set fat concentrations during phantom construction (Fig. 2b, Table 3) as also supported by the slope of 0.954 (coefficient of determination [*R*_2_] = 0.988) of the regression line (Fig. S1). The gradation of PDFFs ranges from 4.4% ± 0.6% in I1 to 17.7% ± 1.4 in I3. Regardless of the iron concentration, the fat fraction remains approximately constant at 20% for I7–I9. The *R*_2_^*^ values increase with higher iron and fat concentration ranging from 24.2 ± 2.1 s^−1^ for the lowest fat concentration (I1) to 211.3 ± 18.8 s^−1^ for the highest fat and iron particle concentration (I9). Iron-only spheres exhibit lower *R*_2_^*^ as those mixed with 20% peanut oil (Fig. 2c). *R*_2_ increases with higher iron concentration (Fig. 2e) from 25.2 ± 0.7 s^−1^ (I4) to 33.9 ± 1.2 s^−1^ (I6). *R*_2_ remained approximately constant at 15 s^−1^ in the fat-only spheres (I1—I3), regardless of the respective fat concentration, and at approximately 21 s^−1^ for I7–I9. *R*_1_ varies between 2.4 ± 0.1 and 2.9 ± 0.2 s^−1^ and decreases with increasing concentrations of fat. The proton MR spectrum acquired in sphere I3 (Fig. 2d) shows six peaks originating from lipids and one at 4.8 ppm originating from water protons. The four labeled peaks were quantifiable reliably and considered for the multi-peak fat model (Fig. 2f). The close proximity to the water peak and a low amplitude limited the evaluability of the methine (–CH = CH–) and methylene (–CH2–COO) peaks, respectively. The manganese phantom is comprehensively characterized in the supplementary material (Fig. S2, Table S1).

**Table 3:** Means and standard deviations of relaxation rates (*R*_1_, *R*_2_, *R*_2_*) and PDFF measured in volumes of interests of the individual spherical inclusions of the iron phantom. The known iron nanoparticle and fat concentrations are shown as comparison. The label of the sample refers to the ones in Fig. 1f, with BG additionally referring to a VOI in the large agar sphere

Sample	*R*_1_ [s^−1^]	*R*_2_ [s^−1^]	*R*_2_^*^ [s^−1^]	PDFF in %	Fe_3_O_4_ [mM]	Fat [vol %]
I1	3.0 ± 0.2	15.5 ± 0.4	24.2 ± 2.0	4.4 ± 0.6	0	4
I2	2.7 ± 0.1	15.6 ± 0.2	30.5 ± 2.0	9.9 ± 0.7	0	10
I3	2.4 ± 0.1	15.0 ± 0.3	40.6 ± 3.3	17.7 ± 1.4	0	20
I4	2.5 ± 0.2	25.2 ± 0.7	94.2 ± 7.2	0.7 ± 0.6	0.13	0
I5	2.4 ± 0.3	29.7 ± 1.0	132.7 ± 6.7	1.0 ± 0.8	0.19	0
I6	2.4 ± 0.3	33.9 ± 1.2	169.3 ± 8.7	1.0 ± 0.9	0.26	0
I7	2.7 ± 0.3	20.6 ± 0.6	127.0 ± 11.5	19.6 ± 5.7	0.13	20
I8	2.9 ± 0.3	21.0 ± 0.6	168.9 ± 13.2	21.3 ± 2.2	0.19	20
I9	2.8 ± 0.3	21.3 ± 0.6	211.3 ± 18.9	21.2 ± 2.2	0.26	20
BG	2.8 ± 0.1	16.3 ± 0.2	20.8 ± 2.1	0.52 ± 0.1	0	0

## Local field and susceptibility map analysis

**Fig. 3:**
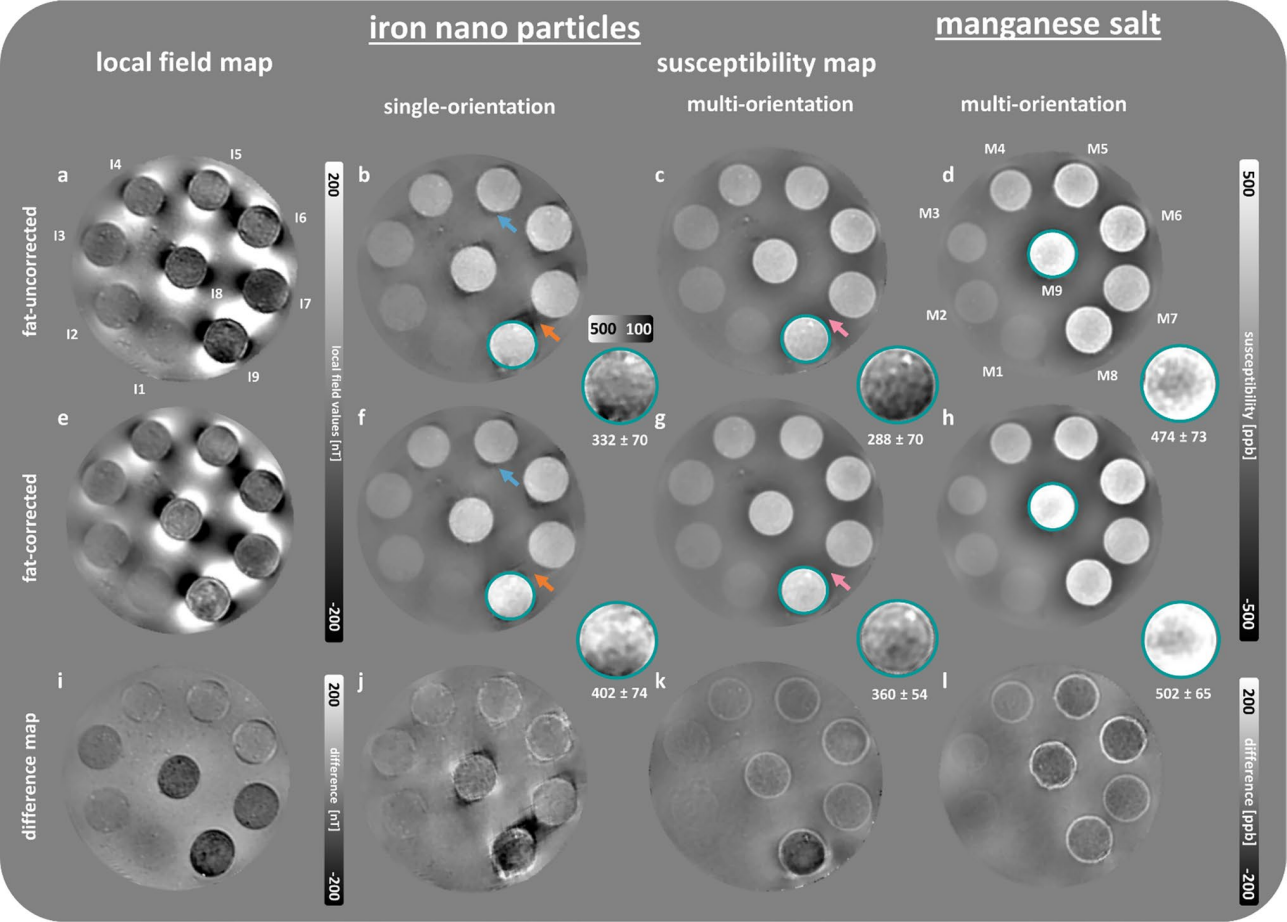
Local field (**a**, **e**) and susceptibility maps (**b**, **c**, **f**, **g**) of a representative slice of the iron phantom and exemplary susceptibility maps of the manganese phantom (**d**, **h**). Fat-uncorrected maps are presented in (**a**–**d**), followed by those experiencing fat correction (**e**–**h**), and the respective difference maps (**i**–**l**) (fat-corrected map subtracted from fat-uncorrected map). Single-orientation field-to-susceptibility inversion was conducted using HEIDI, whereas COSMOS was used for the multi-orientation approach. Enlarged views of the spherical inclusion I9, circled in turquoise and scaled between 100 and 500 ppb, highlight the changes in susceptibility due to fat correction. The mean value and standard deviation of the susceptibilities within the turquoise region are located under the close-up in ppb. All susceptibility maps are referenced to the background agar susceptibility. Orange arrows indicate a reduction of streaking (extraspherical contributions) after fat correction. In the COSMOS maps (pink arrows), these extraspherical contributions are marginal and barely change due to fat correction. The labeling of the small spherical inclusions is provided in (**a**, **d**)

The local field and susceptibility map of the iron phantom are shown in Fig. 3. As expected, the use of fat correction substantially alters the local field values, especially in spheres containing a mixture of iron nanoparticles and fat (I7–I9), which is further supported by the difference map (Fig. 3i). The susceptibility maps are homogeneous in the small spheres’ surrounding agar, but exhibit particularly pronounced extraspherical contributions for spheres containing increasing concentrations of iron nanoparticles. The susceptibility values increase in fat- and iron-only spheres with higher fat and iron particle concentrations. Similarly, the susceptibilities increase with higher iron particle concentrations in the spherical inclusions with 20% fat. Substantial dipole inversion artifacts (Fig. 3, orange arrows) are visible around spheres I7–I9 without fat correction that attenuate with fat correction. As shown in the close-ups of I9 (Fig. 3, turquoise circle), fat correction leads to higher susceptibilities. The average local field and susceptibility values and respective standard deviations from VOI-based analysis are presented in Table S2. We further investigated the suitability of MnCl_2_ as a paramagnetic substance for phantom construction (Fig. 3d, h). The principal findings observed for the phantom containing iron nanoparticles could be replicated with the manganese phantom as well and are presented in more detail in the supplementary materials (Fig. S3, Table S3).

**Fig. 4:**
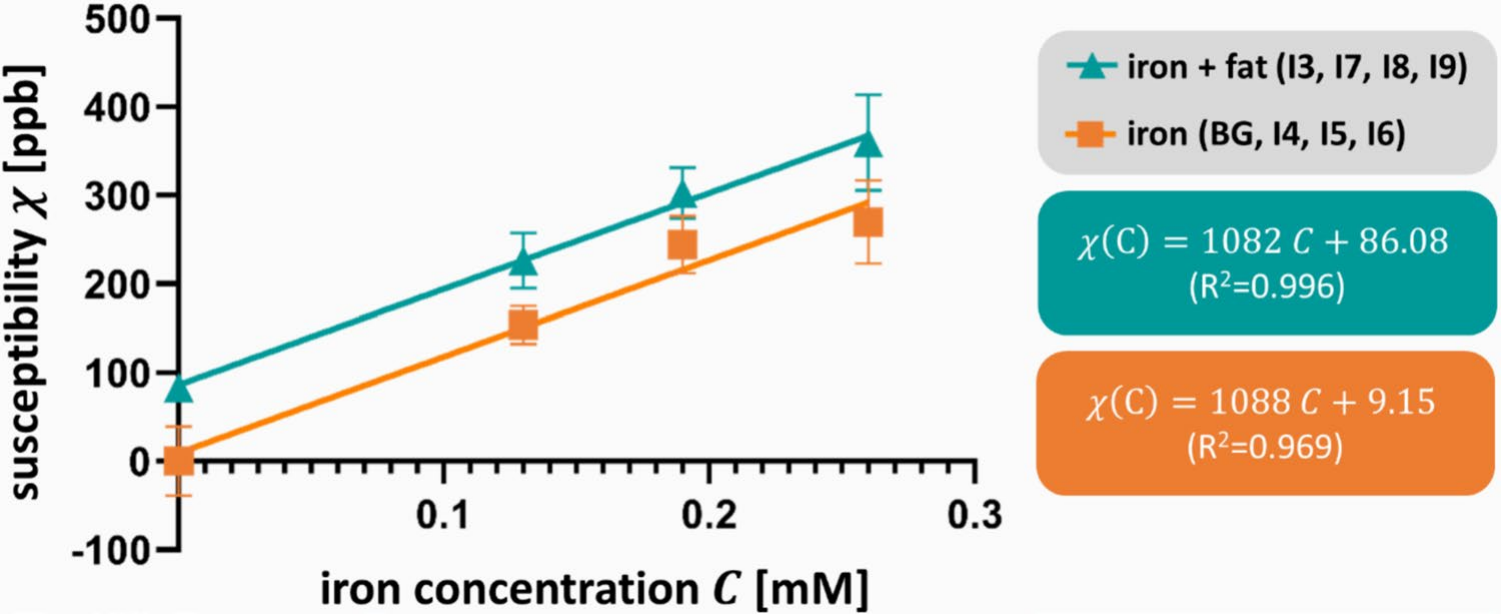
Susceptibility $\chi$ versus iron concentration $\text{C}$ for iron-laden spheres with 20% fat (turquoise) and without fat (orange). BG, background, denotes the volume of interest placed in the large agar sphere. The average susceptibility values and the standard deviations (error bars) of the spherical inclusions are plotted for the COSMOS approach. A linear least squares fit was applied to the data, producing the presented slope, intercept, and coefficient of determination (*R*^2^)

Linear fitting of the COSMOS susceptibilities obtained from fat-corrected field maps as a function of iron concentration (Fig. 4) yielded slopes that were very similar when focusing on pure iron (slope: 1088 ppb/mM) and focusing on iron–fat mixtures (slope: 1082 ppb/mM). The intercept, however, increased substantially from 9.15 ppb (without fat) to 86.08 ppb when fat was present.

## Susceptibility and *R*_2_* analysis

**Fig. 5:**
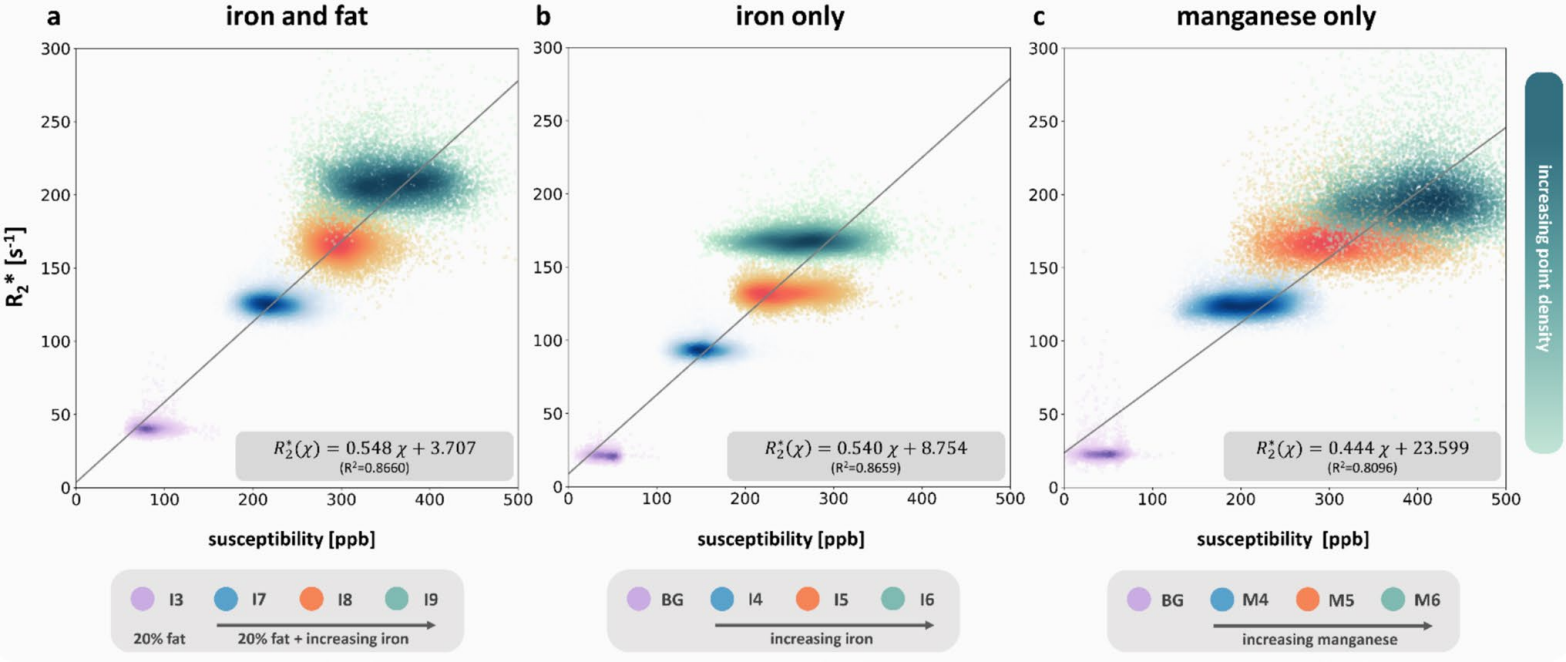
Susceptibility versus *R*_2_* in the presence of fat and iron (**a**), iron only (**b**), and manganese only (**c**). The COSMOS susceptibilities are plotted against the *R*_2_* values for four distinct VOIs. Purple indicates the fat-only VOI (I3) or background VOIs (BG), while blue, orange, and turquoise represent VOIs with increasing concentrations of the paramagnetic substances. The density of data points within each VOI is visualized through color fading. A linear least squares fit was applied to the data, producing the presented slope, intercept, and coefficient of determination (*R*^2^) in the bottom right corner of each plot

The relationship between magnetic susceptibility and *R*_2_* within inclusions of iron, iron and fat, as well as manganese is illustrated in Fig. 5. Overall, the data is more scattered along the susceptibility axis than along the *R*_2_* axis. Both, *R*_2_* and susceptibility, are elevated in spheres containing iron and fat mixtures (Fig. 5a) compared to the ones with iron only (Fig. 5b). Here, the slopes of the fitting line were very similar, with values of 0.548 ppb^−1^*s^−1^ versus 0.540 ppb^−1^*s^−1^, respectively. The values in the inclusions with manganese only (Fig. 5c) are more scattered than the ones observed in the iron-only inclusions. Interestingly, the susceptibilities in M6 are substantially higher than those in I6, while *R*_2_* in M6 is slightly reduced compared to I6.

## Repeatability and long-term stability

**Fig. 6:**
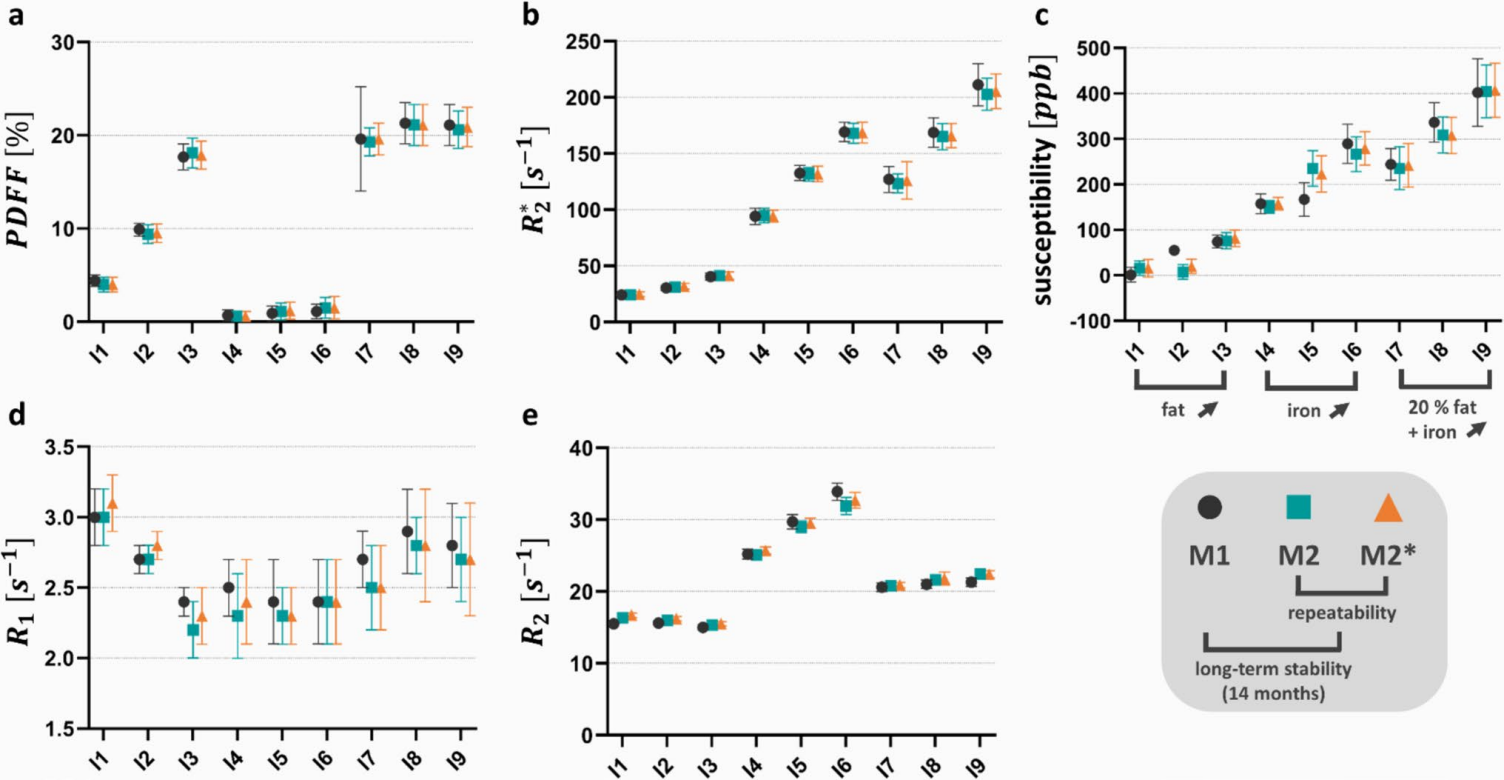
Repeatability and long-term stability assessment. Average PDFF (**a**), *R*_2_* (**b**), susceptibility (**c**), R_1_ (**d**), and *R*_2_ values (**e**) for each spherical inclusion are plotted across different measurement sessions (M1, M2, M2*). Error bars indicate the standard deviation. The repeatability measurements (scan–rescan) M2 (turquoise) and M2* (orange) were conducted 14 months after the initial measurement M1 (dark gray). The susceptibilities were derived from a single-orientation susceptibility map reconstructed using HEIDI-based field-to-source inversion

Figure 6 summarizes the measured relaxation rates (*R*_1_, *R*_2_, *R*_2_*), PDFFs, and magnetic susceptibilities from the initial measurement and two follow-up sessions conducted on the same day after 14 months. The quantitative parameters exhibit similar variations across both time intervals. One-way ANOVA with repeated measurements indicated no statistically significant differences in the average *R*_2_, *R*_2_*, PDFF and susceptibilities across the individual measurements (*p* < 0.05). While ANOVA indicated statistically significant *R*_1_ variations across measurements, these lie within the respective standard deviations.

## Discussion

This work proposed a dedicated phantom design, using agar spheres doped with varying concentrations of paramagnetic solution and fat surrounded in agar, to achieve an interface-free phantom, simulating liver tissue under heathy and pathologic conditions. The phantom was comprehensively characterized regarding its relaxation properties and its fat composition. Additionally, the phantom-specific fat spectrum was obtained by MR spectroscopy for subsequent fat correction. We employed the commonly used [33, 35] iterative graph-cut approach from Hernando et al. [46] to correct the SHARP-processed local field maps for the influence of fat.

The analysis of susceptibility maps demonstrated the influence of fat correction, particularly in regions near inclusions with iron–fat mixtures, where artifacts, notably high-frequency streaking, were markedly reduced on fat-corrected maps of the single-orientation approach (Fig. 3, orange arrows). Because of the intrinsic oversampling of the field-to-susceptibility inversion problem in COSMOS, extra-spherical artifacts were less pronounced than on the single-orientation susceptibility maps even in particular if the local field is not fat corrected. We generally noticed increased susceptibility values in iron- and manganese-laden spheres after fat correction for both the single- and multi-orientation approach.

As expected, we observed a linear relationship between susceptibility and iron concentration, with slopes representing the molar susceptibility at 3T that were similar in both fat-free and fat-containing inclusions. The slope of 1088 ppb/mM in fat-free inclusions (Fig. 4) was substantially higher compared to that in other phantom studies. For instance, Dietrich et al. [64] reported a slope of 88 ± 3 ppb/mM for ferric chloride (FeCl_3_) solutions, and Gustavo Cuna et al. [65] found slopes of 161 ± 32 ppb/mM for free iron and 40 ± 2 ppb/mM for clustered iron (iron absorbed in sodium polyacrylate), suggesting that differences in dopants, such as our use of super-paramagnetic iron oxide particles with core sizes of 4–6 nm as measured by transmission electron microscopy, may account for the observed deviations. Our slope is very close to that of monocrystalline iron oxide nanoparticles with the same core size (MION-46L) for which a molar susceptibility of 1382.3 ppb/mM has been reported [66, 67]. To our best knowledge, there is no study comparing liver susceptibility with biopsy-based iron measurements. However, in some studies, in vivo liver susceptibility was correlated to LIC measured via the biopsy-calibrated FerriScan technique yielding slopes of approximately 100–200 ppb*g dw/mg (slopes were estimated from the diagrams). After converting dry weight to wet weight using the conversion factor 0.2439 (1/4.1) [35, 68], and dividing it by the molar mass of iron (55.85 g/mol), these slopes were 436 ppb/mM and 873 ppb/mM. Despite the substantially smaller particle size we used to that in the liver (0.1–3.2 µm) [69], we observed a relationship between susceptibility and iron in a similar range to that determined in vivo.

In contrast to the slope in Fig. 4, our linear regression between susceptibility and iron concentration revealed markedly different intercepts for fat-free inclusions (*i*_ff_ = 9.2 ppb) and fat-containing inclusions (*i*_fc_ = 86.1 ppb). This difference is expected, since fat has a slightly higher magnetic susceptibility (susceptibility of protons in fat: − 8.44 ppm) than water (${\chi }_{{\text{H}}_{2}\text{O}}$ = − 9.05 ppm) [70]. Clinically, however, such differences are critical. Using our regression equations (Fig. 4), a susceptibility of 200 ppb translates to 0.175 mM iron in fat-free tissue, but only 0.105 mM iron in tissue with 20% fat—a reduction of approximately 40%. Consequently, fat concentration must be accounted for by establishing a calibration curve between magnetic susceptibility and PDFF. Our data show a strong linear relationship between susceptibility and measured PDFF (${\chi }_{\text{fat}}\left(\text{PDFF}\right)=4.83\frac{\text{ppb}}{{\%}}\cdot \text{PDFF}\%-6.49 \text{ppb}$, *R*^2^ = 0.91, determined in the inclusions: I1, I2, I3, the surrounding medium). Incorporating this correction into the regression model without fat content (${c}_{\text{Fe}}\left(\chi , \text{PDFF}\right)=\left(\chi -{i}_{ff}-{\chi }_{\text{fat}}\left(\text{PDFF}\right)\right)/1088 \text{ppb}/\text{mM}$), a 200 ppb shift translates to an estimated iron concentration of 0.093 mM at 20% fat and 0.026 mM at 35% fat. As susceptibility-based iron quantification becomes less accurate with increasing fat content, its reliability may be compromised in patients with fatty liver, especially those with severe steatosis. Similar issues have been reported for *R*_2_*-based liver iron quantification, where correcting *R*_2_* values for PDFF effects has been proposed [71]. Overall, further research is required to explore the relationship between magnetic susceptibility and PDFF and develop a fat-compensated approach for iron quantification.

Linear regression analyses confirm that both susceptibility and *R*_2_* are linear indicators of iron content, suggesting that higher iron concentrations are often associated with magnetic field inhomogeneities at the voxel level. The slopes of the linear relationship between *R*_2_* and COSMOS susceptibility observed in this study (iron only: 0.54, iron and fat: 0.548) were notably higher than those reported in the liver in vivo using anisotropic GRE-VIBE imaging (Sharma et al. 2015 [35]: 0.357 s/ppb, Sharma et al. 2017 [34]: 0.294 s/ppb, Li et al. [19]: 0.333 s/ppb), likely due to differences between phantom and in vivo conditions, such as the homogeneous iron storage in the phantom versus ferritin cores in the liver, the smaller iron particle sizes (4–6 nm) compared to the ones in the liver (0.1–3.2 µm) [69], or different acquisition and post-processing protocols. In comparison, slopes between *R*_2_* and susceptibility in cerebral gray matter ranged from 0.117 to 0.157 ppb⁻^1^ s⁻^1^ (adjusted from 7 to 3T) [72, 73], with smaller slopes potentially attributed to reduced intravoxel spin dephasing due to smaller voxel sizes. It is important to note that the fundamental mechanisms underlying susceptibility changes and their effects on MRI signals do not imply a direct relationship between magnetic susceptibility and *R*_2_*. While pure susceptibility differences induce a frequency shift of the Larmor frequency without affecting *R*_2_*, intravoxel susceptibility variations increase intravoxel dephasing, hence elevating *R*_2_* [73, 74]. Thus, susceptibility-based estimation of iron concentration is insensitive to the microscopic spatial distribution of iron, potentially providing a distribution independent measure of iron concentration.

The phantoms we constructed met the specified criteria and showed their suitability through a thorough characterization process. Visual appraisal revealed that the assembled phantom is made of homogeneously distributed solutions within the larger and smaller spheres that contain negligible amounts of air inclusions. The presence of air bubbles must be minimized, as they introduce susceptibility differences of about 10 ppm [20] at the air–liquid interfaces, leading to local magnetic field distortions. These distortions can cause phase wrapping artifacts, signal dephasing in gradient echo sequences, and inaccuracies in QSM. Additionally, air inclusions may interfere with water–fat separation algorithms and bias *R*_2_* measurements. The repeatability and long-term stability assessments demonstrated consistent quantitative parameters across sessions and a 14-month follow-up, suggesting that the phantom maintains its stability over at least this time period. The stability is likely facilitated by the phantom’s storage in an airtight container at room temperature, which prevents desiccation and preserves the integrity of its constituents. These results support the phantom’s potential as a reliable reference for evaluating new MRI approaches in longitudinal MRI studies, though further investigations over longer time frames could provide additional insights into its durability.

The quantitative analysis yielded values within the range of liver tissue, and the susceptibility maps further confirmed the feasibility of the spherical phantom design. To ultimately bridge the gap between the phantom signal and the in vivo liver, several adaptations are conceivable to overcome the limitations of the current phantom design. Firstly, while the phantoms replicate paramagnetic and fat-based signal contributions, the current concentrations achieve maximal *R*_2_* values, which are indicative of mild pathological iron overload [41]. MRI fat fractions and the grading of steatosis, which measures the percentage of cells with intracellular fat vacuoles, do not directly correlate [75]. Nonetheless, Tang et al. [51] established thresholds for steatosis grading, allowing the phantom to simulate steatosis grades 0, 1 (PDFF < 6.4% to < 17.4%), and 2 (PDFF < 22.1%). When increasing fat concentrations in future phantoms, strategies to prevent the formation of inhomogeneous agar solutions for fat concentrations over 20% have to be considered. Here, incorporating both water-soluble and oil-soluble surfactants during phantom construction could stabilize the water–agar–fat emulsion more effectively [76]. Other Potential confounders—including Imprägnol and 1% formaldehyde—were carefully managed to maintain the phantom's structural integrity. Due to the application of Imprägnol only at the surface of the small spherical inclusion, any impact on quantitative MR parameters measured within a rather large VOI is expected to be negligible. As formaldehyde was uniformly added to all solutions of the inclusions, its influence, which at the applied concentration, is substantially lower than that of the other compounds (iron, fat, MnCl₂) [77], remains consistent across all inclusions and does not affect the analysis of their varying concentrations. Secondly, our proposed phantom does not specifically account for liver fibrosis, which alters T_1_ values. This limitation could be addressed by incorporating nickel chloride, as demonstrated by Zhao et al. [39]. Given that nickel chloride introduces a distinct paramagnetic susceptibility contribution (molar susceptibility of ${\chi }_{\text{NiCl}2}$ at 20 °C = 4436 * 10^–6^ ml/mol) [78], its impact on the phantom’s overall susceptibility has to accounted for by adjusting the concentration of the paramagnetic solution during construction. Thirdly, on a microscopic level, the composition of the phantom and liver tissue differs. In liver tissue, iron ions are stored in ferritin shells within hepatocytes [7, 8], and fatty acids that accumulate as intrahepatocytic lipid vesicles, i.e., lipid droplets in the cytoplasm of hepatocytes, with a range of droplet sizes [79, 80]. In our phantom, however, iron nanoparticles are mixed directly into the agar–fat solution, likely causing iron particles to segregate into the agar–water phase while the hydrophobic lipids from peanut oil form globules. Although the use of surfactants might alleviate this effect at higher fat concentrations (> 20%), we assume a macroscopically homogeneous distribution of lipids and iron particles. Nevertheless, the question remains whether the droplet size distribution in the phantom matches that observed in hepatocytes. In vivo, droplet sizes vary with fat volume fraction, where smaller droplets dominate at lower fat fractions, while coalescence at higher fractions yields larger droplets. In contrast, the phantom’s mixing process may yield a relatively uniform droplet size regardless of the fat concentration. To assess and compare the droplet sizes within the phantom with those reported in liver tissue, microscopy (light or electron microscopy) or laser diffraction techniques [81] would be straightforward approaches. Recently, a study for estimating lipid droplet sizes in adipose tissue using MRI was proposed [82], representing an MR technique to potentially estimate our phantom’s droplet sizes.

We demonstrated that manganese chloride can also be used as paramagnetic substance to replicate the *R*_2_* and susceptibility effects caused by iron accumulation. Despite the lower cost of manganese chloride compared to iron nanoparticles, it exhibits different relaxivities [39, 83], which influences the quantity of the chemical required to achieve the desired relaxation rates. Moreover, Zhao et al. [39] showed that manganese chloride solely alters *R*_2_* of water, whereas iron microspheres affect the *R*_2_* of both water and fat. Other chemicals mimicking effects of LIC on MRI are also conceivable. For instance, the combination of paramagnetic salt with iron microspheres has been shown to provide more realistic signal behaviors, closer to those of in vivo measurements, as shown by relaxometry studies [39, 84, 85]. Therefore, it would be worthwhile to explore such a configuration in spherical inclusions of the proposed phantom in future studies.

## Supplementary Information

Below is the link to the electronic supplementary material.

Supplementary file1 (DOCX 1435 KB)

## Data Availability

The data supporting this study’s findings are available from the corresponding author upon reasonable request.
