# Supplementary material for: A dedicated phantom for exploring the interplay of fat and paramagnetic substances in quantitative susceptibility mapping
Source: MAGMA. 2025 Jun 2;38(6):905–20. doi: 10.1007/s10334-025-01261-3 (PMC12638357; doi:10.1007/s10334-025-01261-3)
Supplement: Supplementary file 1 — Supplementary file1 (DOCX 1435 KB) [file 10334_2025_1261_MOESM1_ESM.docx]

Supplementary Material.

Theoretical considerations on phase and susceptibility.

The gradient echo phase $\phi\left( \vec{r}, t \right)$ at the spatial location vector $\vec{r}$ and echo time $t$ is given by:

|  | $\phi\left( \vec{r}, t \right)= \phi_{0}\left( \vec{r} \right)+ 2\pi\Delta f\left( \vec{r} \right)t$*,* | (A.1) |
| --- | --- | --- |

where $\phi_{0}(\vec{r})$ is a time-independent phase offset comprising static phase contributions related to the radiofrequency transmission and signal reception processes [44] and $\Delta f\left( \vec{r} \right)$ denotes the measured frequency variation. With the local Larmor frequency $f_{L}$, the demodulation frequency $f_{R}$ and the magnetic field $B\left( \vec{r} \right) =(B_{0}+B_{inh}\left( \vec{r} \right)+B_{\chi}\left( \vec{r} \right))(1-\sigma(\vec{r}))$ Eq. A.1 may be rewritten [22]:

|  | $\phi\left( \vec{r}, t \right)= \phi_{0}\left( \vec{r} \right)+2\pi\left( f_{L}\left( \vec{r} \right)-f_{R} \right)t$ | (A.2) |
| --- | --- | --- |
|  | ${=\phi}_{0}\left( \vec{r} \right)+2\pi\left( \frac{\gamma}{2\pi}B\left( \vec{r} \right)- f_{R} \right)t$ |  |
|  | ${= \phi}_{0}\left( \vec{r} \right)+2\pi\left( \frac{\gamma}{2\pi}\left( B_{0}+B_{inh}\left( \vec{r} \right)+B_{\chi}\left( \vec{r} \right) \right)\left( 1-\sigma\left( \vec{r} \right) \right)- f_{R} \right)t$ |  |

Here, $B_{0}$ denotes the nominal magnetic field strength generated by the main magnet under ideal conditions, that is, in the absence of any material or subject within the scanner that could alter the field homogeneity. $B_{inh}\left( \vec{r} \right)$ and $B_{\chi}\left( \vec{r} \right)$ denote magnetic field contributions arising from intrinsic inhomogeneities and the magnetic susceptibility $\chi$, respectively. Equation A.2 can be rearranged and simplified using the following assumption. Since $B_{0}\gg B_{inh}\left( \vec{r} \right)+B_{\chi}\left( \vec{r} \right)$ and $B_{0}$ is assumed to be constant within the field-of-view, the corresponding frequency is $f_{0}=\frac{\gamma}{2\pi}B_{0}$. The measurement is conducted relative to the pre-adjusted demodulation frequency $f_{R}$, which is typically close to $f_{0}$, but not equal. With a correctly adjusted demodulation frequency ($f_{R} \approx f_{0}$) both components cancel out. In line with QSM literature [22, 43] and for clarity, this small frequency difference is typically omitted (Eq. A.3, $\dot{\approx}$).

|  | $\phi\left( \vec{r}, t \right)=\phi_{0}\left( \vec{r} \right)+2\pi\left( \frac{\gamma}{2\pi}\left( B_{0}+B_{inh}\left( \vec{r} \right)+B_{\chi}\left( \vec{r} \right) \right)\left( 1-\sigma(\vec{r}) \right)-f_{R} \right)t$ | (A.3) |
| --- | --- | --- |
|  | $= \phi_{0}\left( \vec{r} \right)+2\pi\left( \frac{\gamma}{2\pi}\left( B_{inh}\left( \vec{r} \right)+B_{\chi}\left( \vec{r} \right) \right)\left( 1-\sigma\left( \vec{r} \right) \right)+(\frac{\gamma}{2\pi}B_{0}(1-\sigma(\vec{r})){- f}_{R}) \right)t$ |  |
|  | $= \phi_{0}\left( \vec{r} \right)+2\pi\left( \frac{\gamma}{2\pi}\left( B_{inh}\left( \vec{r} \right)+B_{\chi}\left( \vec{r} \right) \right)\left( 1-\sigma(\vec{r}) \right)-\frac{\gamma}{2\pi}B_{0}\sigma(\vec{r})+(f_{0}{- f}_{R}) \right)t$ |  |
|  | $\dot{\approx}\phi_{0}\left( \vec{r} \right)+\gamma\left( (B_{inh}\left( \vec{r} \right)+B_{\chi}\left( \vec{r} \right))(1-\sigma\left( \vec{r} \right))-B_{0}\sigma(\vec{r}) \right)t$ |  |

With respect to Eq. 1, $\sigma$ denotes the spectral peak area-weighted average chemical shift in a voxel that can be defined as $\sigma=\bar{f_{F}}$ and $f_{B}\left( \vec{r} \right)= \frac{\gamma}{2\pi}(B_{inh}\left( \vec{r} \right)+B_{\chi}\left( \vec{r} \right))$. It follows:

|  | $\phi\left( \vec{r}, t \right)= \phi_{0}\left( \vec{r} \right)+\gamma\left( (\frac{2\pi}{\gamma}f_{B}(\vec{r}))(1-\sigma\left( \vec{r} \right))-B_{0}\sigma(\vec{r}) \right)t$. | (A.4) |
| --- | --- | --- |
|  | $\phi\left( \vec{r}, t \right)= \phi_{0}\left( \vec{r} \right)+\gamma\left( \frac{2\pi}{\gamma}f_{B}\left( \vec{r} \right)-\frac{2\pi}{\gamma}f_{B}\left( \vec{r} \right)\sigma\left( \vec{r} \right)-B_{0}\sigma(\vec{r}) \right)t$. |  |

The chemical shift is on the order of parts-per-millions (ppm, chemical shift between water and fat: 3.5 ppm [45]) and can locally affect the phase substantially if not corrected. While the chemical shift is generally assumed to be spatially constant in the brain, this assumption does not hold for fat-containing tissues in regions such as the abdomen or in our proposed phantom. For fat-containing samples, chemical-shift encoded reconstruction incorporating fat correction [46,47,48] can be applied, to remove the phase contributions arising from the chemical shift. Following fat correction, the field contribution due to the spatially dependent chemical shift, $\sigma\left( \vec{r} \right)$, is largely eliminated, with only a small, uniform residual frequency error remaining, resulting from imperfect modeling of the multi-peak fat spectrum, that can be approximated as a constant,$f_{err}$ (A.5, $\ddot{\approx}$). Since $f_{err}$ is expected to be a few Hz, its impact is negligible, leading to the following approximation (A.5, $\approx$):

|  | $\phi\left( \vec{r}, t \right)\ddot{\approx} \phi_{0}\left( \vec{r} \right)+\gamma\left( (\frac{2\pi}{\gamma}f_{B}(\vec{r})-f_{err}) \right)t$ | (A.5) |
| --- | --- | --- |
|  | $\phi\left( \vec{r}, t \right)\approx\phi_{0}\left( \vec{r} \right)+\gamma\left( (\frac{2\pi}{\gamma}f_{B}(\vec{r})) \right)t$ |  |
|  | $\phi\left( \vec{r}, t \right)\approx\phi_{0}\left( \vec{r} \right)+\gamma\left( B_{inh}\left( \vec{r} \right)+B_{\chi}\left( \vec{r} \right) \right)t$. |  |

Based on equation A.4 and A.5, and in the case of appropriate shimming ($B_{inh}\left( \vec{r} \right)\ll B_{\chi}(\vec{r})$), the magnetic field originating from magnetic sources within a given volume of interest (VOI) $B_{\chi, int}(\vec{r})$ may be obtained after removing magnetic field contributions arising from magnetic sources outside that VOI $B_{\chi, ext}(\vec{r})$ (e.g., using SHARP):

|  | $\tilde{B}\left( \vec{r} \right)= B_{inh}\left( \vec{r} \right)+B_{\chi}\left( \vec{r} \right)$ | (A.6) |
| --- | --- | --- |
|  | $=B_{inh}\left( \vec{r} \right)+B_{\chi,ext}\left( \vec{r} \right)+B_{\chi,int}\left( \vec{r} \right)$ |  |
|  | $\approx B_{\chi,int}\left( \vec{r} \right)\approx\left( B_{0} \left( \chi\left( \vec{r} \right)⊛d_{z}\left( \vec{r} \right) \right) \right).$ |  |

Hence, the local field observed due to the tissue within the specified VOI is given by the magnetic susceptibility distribution $\chi\left( \vec{r} \right)$, where $d_{z}\left( \vec{r} \right)$ refers to the unit dipole response (Eq. A.6, third line).

Characterization of the manganese phantom.

The manganese phantom is characterized with parametric maps and VOI-based analysis in Supplementary Fig. 1 and Supplementary Tab. 1. Similar to the iron phantom, the high-resolution GRE-VIBE magnitude image (Supplementary Fig. 1 a) shows a homogenous background with a few speckles indicating air bubbles. The PDFFs correspond to the set values and range from 5.20 ± 0.59 in M1 to 22.07 ± 1.41 in M3 and remains constant around 21% for M7 – M9. The R2* values increase with increasing manganese and fat concentration from 26.90 ± 2.45 s−1 (M1) to 228.19 ± 28.88 s−1 (M9). R2* is elevated in spheres containing both manganese and fat (M7 – M9) compared to the manganese only spheres (M4 – M6). R2 increases with increasing manganese concentration and remains approximately constant at 16 s−1 in the fat-only spheres (M1—M3), regardless of the respective fat concentration, and at approximately 20 s−1 for M7 – M9. R1 varies between 2.28 ± 0.22 s−1 and 2.66 ± 0.27 s−1 and decreases with increasing concentrations of fat.


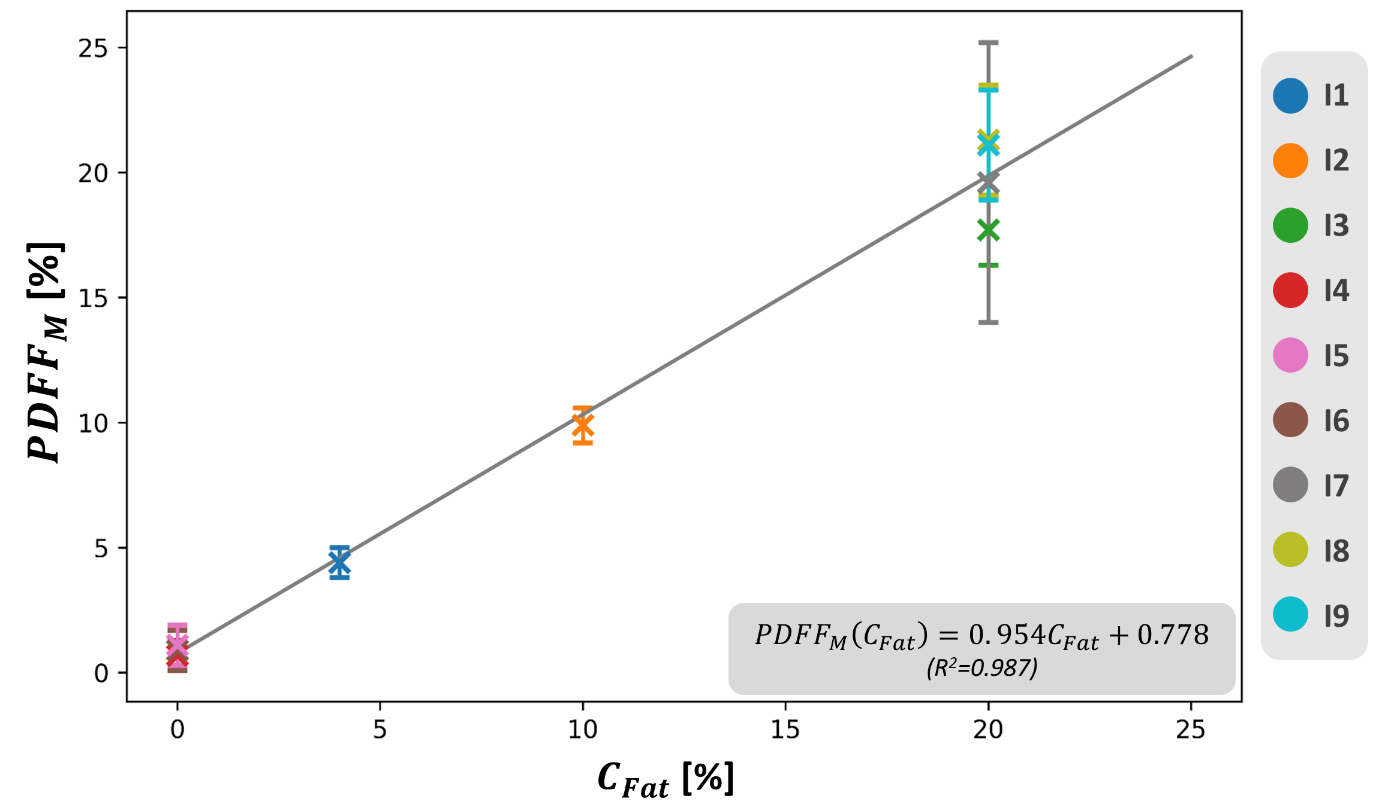


**Supplementary Fig. 1** Known fat concentration $c_{Fat}$ vs. measured PDFF (PDFF_M_) values is presented for the spheres I1 - I9 in individual colors. Error bars indicate the standard deviation of the measured PDFF. A linear least squares fit was applied to the data, producing the slope, intercept, and coefficient of determination (R²) presented in the lower right corner.

**
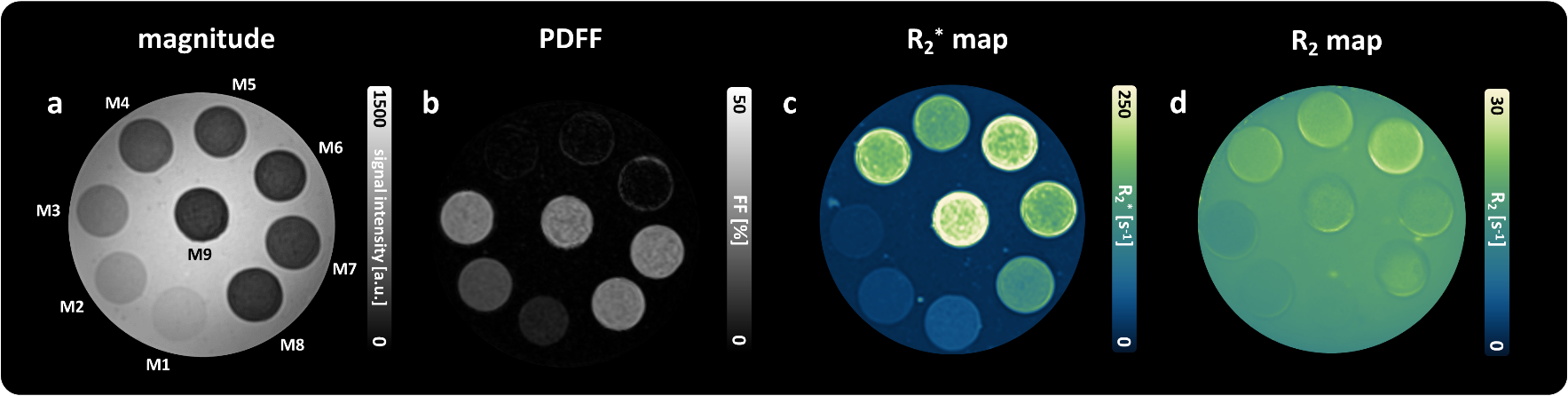
Supplementary Fig. 2** Comprehensive characterization of the manganese phantom. One representative slice of the GRE-VIBE magnitude image at TE=6.3 ms, the PDFF map, the $R_{2}^{*}$ map and R_2_ map are presented in (a) to (d), respectively. The small spheres are exemplary labeled in (a)

**
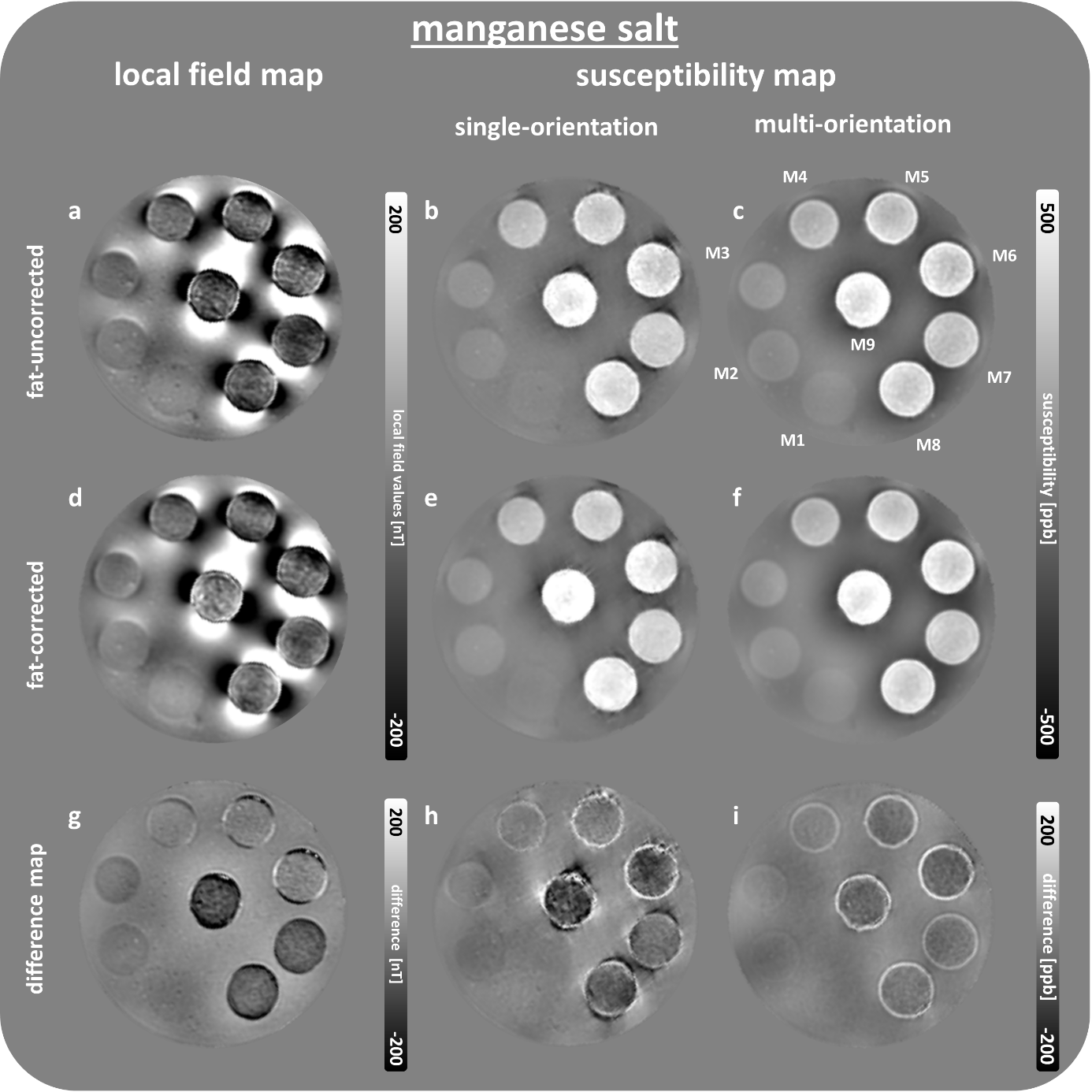
**

**Supplementary Fig. 3** Local field and susceptibility maps of a representative slice of the manganese phantom. Fat uncorrected maps are presented in (a-c), followed by those experiencing fat correction (d-f), and the respective difference maps (g-i) (fat-corrected map subtracted from fat uncorrected map). Single-orientation field-to-susceptibility inversion was conducted using HEIDI, whereas COSMOS was used for the multi-orientation approach. All susceptibility maps are referenced to the background agar susceptibility. The labeling of the small spherical inclusions is provided in (c)

**Supplementary Table 1** Means and standard deviations of relaxation rates (R_1_, R_2_, R_2_*) and PDFFs measured in volumes of interests of the individual spherical inclusions of the manganese phantom. The known iron nano particle and fat concentrations are shown as comparison. The label of the sample refers to the ones in Fig. 1g

| sample | R_1_ [s^-1^] | R_2_ [s^-1^] | R_2_^*^ [s^-1^] | PDFF in % | MnCl_2_ [mM] | Fat [vol%] |
| --- | --- | --- | --- | --- | --- | --- |
| M1 | 2.6±0.2 | 16.6±0.3 | 26.9±2.5 | 5.2±0.6 | 0 | 4 |
| M2 | 2.5±0.1 | 16.2±0.3 | 36±1.9 | 12.6±0.7 | 0 | 10 |
| M3 | 2.3±0.1 | 15.6±0.3 | 50.5±2.9 | 22.1±1.4 | 0 | 20 |
| M4 | 2.4±0.2 | 20±0.4 | 126.8±9.9 | 1.3±0.9 | 2.5 | 0 |
| M5 | 2.3±0.2 | 20.8±0.7 | 173.6±17.0 | 1.8±1.3 | 3.5 | 0 |
| M6 | 2.3±0.2 | 22.4±0.5 | 205.7±31.5 | 1.9±2.1 | 4.5 | 0 |
| M7 | 2.6±0.2 | 19.2±0.4 | 159.3±12.4 | 22.9±2.2 | 2.5 | 20 |
| M8 | 2.7±0.3 | 20±0.2 | 188.5±16.0 | 20.9±5.3 | 3.5 | 20 |
| M9 | 2.7±0.3 | 19.5±0.7 | 228.2±28.9 | 21.3±5.9 | 4.5 | 20 |

**Supplementary Table 2** Means and standard deviations of the local field and susceptibility values in volumes of interests of the individual spherical inclusions of the iron phantom without fat correction (uncorr) and with fat correction (fat corr). Susceptibility values are shown for the single-orientation (HEIDI) and multi-orientation (COSMOS) approach. The susceptibility is referenced to that of the spheres’ surrounding medium and given in parts-per-billion (ppb). The label of the sample refers to the ones in Fig. 1f, with BG additionally referring to a VOI in the large agar sphere

|  | local field map [nT] | | susceptibility [ppb] | | | |
| --- | --- | --- | --- | --- | --- | --- |
| sample | **uncorr** | **fat corr** | **HEIDI**  **uncorr** | **HEIDI**  **fat corr** | **COSMOS**  **uncorr** | **COSMOS**  **fat corr** |
| I1 | -7.8±20.7 | 9.1±19.1 | 13.3±14.5 | 1.4±16.1 | 59.2±14.2 | 43.4±17.0 |
| I2 | -21.4±17.7 | -8.2±13.8 | 53.7±10.1 | 55.0±9.0 | 69.2±13.2 | 56.4±10.5 |
| I3 | -45.7±18.2 | -19.1±12.8 | 72±19.1 | 74.4±14.2 | 76.7±15.7 | 83.4±12.7 |
| I4 | -26.4±18.9 | -27.4±13.6 | 163.2±23.5 | 157.7±21.6 | 162.7±24.2 | 153.5±21.9 |
| I5 | -12.2±31.1 | -11.5±29.2 | 190.6±40.6 | 167.1±37.0 | 231.9±33.2 | 244.4±32.9 |
| I6 | -35.1±38.7 | -43.2±35.6 | 301±44.5 | 289.6±43.1 | 268.5±48.7 | 269.7±47.2 |
| I7 | -99.6±37.4 | -40.6±31.2 | 248±47.2 | 244.1±34.7 | 226.4±33.3 | 226.2±30.9 |
| I8 | -60.8±34.6 | 18.9±38.3 | 307.7±36.5 | 336.6±43.5 | 304.8±31.0 | 302.8±28.7 |
| I9 | -79.7±49.3 | 8.7±43.2 | 358.3±55.9 | 402.3±74.0 | 348.7±57.0 | 359.8±53.8 |
| BG | - | - | 0 ± 29.9 | 0 ± 32.7 | 0 ± 37.3 | 0 ± 39.3 |

**Supplementary Table 3** Means and standard deviations of the local field and susceptibility values in volumes of interests of the individual spherical inclusions of the manganese phantom without fat correction (uncorr) and with fat correction (fat corr). Susceptibility values are shown for the single-orientation HEIDI and multi-orientation COSMOS approach. The susceptibility is referenced to that of the spheres’ surrounding medium and given in parts-per-billion (ppb). The label of the sample refers to the ones in Fig. 1g, with BG additionally referring to a VOI in the large agar sphere

|  | local field map [nT] | | susceptibility [ppb] | | | |
| --- | --- | --- | --- | --- | --- | --- |
| sample | **uncorr** | **fat corr** | **HEIDI**  **uncorr** | **HEIDI**  **fat corr** | **COSMOS**  **uncorr** | **COSMOS**  **fat corr** |
| M1 | 10.8±15.1 | 34.9±16.0 | 31.5±13.7 | 26.3±15.3 | 48.7±15.0 | 57.9±20.0 |
| M2 | -8.5±13.6 | -0.5±11.1 | 58.6±8.9 | 80.1±11.3 | 48.3±14.6 | 64.8±12.2 |
| M3 | -41.8±15.5 | -20.8±16.2 | 74.5±13.1 | 78.4±15.6 | 88.2±18.1 | 81.7±23.6 |
| M4 | -28.1±29.1 | -29.3±26.7 | 235.7±39.4 | 243.6±36.1 | 209±39.8 | 207.1±39.1 |
| M5 | -46.9±59.6 | -42.2±58.8 | 320.2±52.7 | 328±47.3 | 294.2±57.5 | 318.8±60.1 |
| M6 | -35.9±69.6 | -39±64.2 | 397.5±92.6 | 461.2±78.1 | 359.3±72.1 | 404.2±66.3 |
| M7 | -97.6±39.3 | -43±39.6 | 324.7±35.6 | 350.4±37.8 | 285.4±36.4 | 306.9±33.5 |
| M8 | -58.9±42.1 | -5.1±46.3 | 408.3±48.2 | 466.4±50.5 | 357.6±45.8 | 368.7±46.1 |
| M9 | -69.7±63.1 | 25±62.9 | 499.1±88.5 | 576.8±82.5 | 473.9±74.3 | 501.8±65.5 |
| BG | - | - | 0 ± 35.6 | 0 ± 39.7 | 0 ± 52.8 | 0 ± 53.7 |
